# Targeting *N*-Acetylglucosaminidase in *Staphylococcus aureus* with Iminosugar Inhibitors

**DOI:** 10.3390/antibiotics13080751

**Published:** 2024-08-10

**Authors:** Janja Sluga, Tihomir Tomašič, Marko Anderluh, Martina Hrast Rambaher, Gregor Bajc, Alen Sevšek, Nathaniel I. Martin, Roland J. Pieters, Marjana Novič, Katja Venko

**Affiliations:** 1 Laboratory for Cheminformatics, Theory Department, National Institute of Chemistry, Hajdrihova ulica 19, 1000 Ljubljana, Slovenia; janja.sluga@ki.si (J.S.); marjana.novic@ki.si (M.N.); 2 Department of Pharmaceutical Chemistry, Faculty of Pharmacy, University of Ljubljana, Aškerčeva cesta 7, 1000 Ljubljana, Slovenia; tihomir.tomasic@ffa.uni-lj.si (T.T.); marko.anderluh@ffa.uni-lj.si (M.A.); martina.hrast-rambaher@ffa.uni-lj.si (M.H.R.); 3 Department of Biology, Biotechnical Faculty, University of Ljubljana, Jamnikarjeva 111, 1000 Ljubljana, Slovenia; gregor.bajc@bf.uni-lj.si; 4 Department of Chemical Biology & Drug Discovery, Utrecht University, Universiteitsweg 99, 3584 Utrecht, The Netherlandsn.i.martin@biology.leidenuniv.nl (N.I.M.); r.j.pieters@uu.nl (R.J.P.); 5 Biological Chemistry Group, Institute of Biology, Leiden University, Sylviusweg 72, 2333 Leiden, The Netherlands

**Keywords:** autolysin E, glycoside hydrolase, iminosugars, surface plasmon resonance, enzyme inhibition

## Abstract

Bacteria are capable of remarkable adaptations to their environment, including undesirable bacterial resistance to antibacterial agents. One of the most serious cases is an infection caused by multidrug-resistant *Staphylococcus aureus*, which has unfortunately also spread outside hospitals. Therefore, the development of new effective antibacterial agents is extremely important to solve the increasing problem of bacterial resistance. The bacteriolytic enzyme autolysin E (AtlE) is a promising new drug target as it plays a key role in the degradation of peptidoglycan in the bacterial cell wall. Consequently, disruption of function can have an immense impact on bacterial growth and survival. An in silico and in vitro evaluation of iminosugar derivatives as potent inhibitors of *S. aureus* (AtlE) was performed. Three promising hit compounds (**1**, **3** and **8**) were identified as AtlE binders in the micromolar range as measured by surface plasmon resonance. The most potent compound among the SPR response curve hits was **1**, with a *K*_D_ of 19 μM. The *K*_D_ value for compound **8** was 88 μM, while compound **3** had a *K*_D_ value of 410 μM.

## 1. Introduction

Humans have always been exposed to bacterial infections, which are the second-most common cause of death worldwide. Unfortunately, almost one-fifth of all deaths in the human population are still due to bacterial infections, since bacteria are capable of remarkable adaptations to their environment, including undesirable bacterial resistance to antibacterial agents [1]. Severe infections caused by *Staphylococcus aureus* include multidrug-resistant strains that are particularly resistant to methicillin and vancomycin, such as MRSA (methicillin-resistant *S. aureus*) and VRSA (vancomycin-resistant *S. aureus*) [2]. Furthermore, especially in the developed world, MRSA is no longer only a hospital-acquired infection but is, nowadays, also widespread outside hospitals, and *S. aureus* strains resistant to most known antibiotics have been isolated [3]. In general, *S. aureus* can cause a variety of infections, which are classified into several groups depending on pathogenesis and symptoms: localized skin and soft tissue infections, bacteremia, central nervous system infections, upper and lower respiratory tract infections, musculoskeletal infections, urinary tract infections and staphylococcal infections due to toxin exposure [4,5].

With regard to the existing antibiotics, there are several limitations to their use due to the hardy resistance of MRSA/VRSA to beta-lactam antibiotics (methicillin, penicillins, amoxicillin, cephalosporins), carbapenems (imipenem, meropenem), macrolides (erythromycin, clarithromycin), fluoroquinolones (ciprofloxacin and levofloxacin), glycopeptides (teicoplanin), etc. Therefore, it is difficult to treat MRSA/VRSA infections with standard therapies, so the pool of effective antibiotics is very limited, and the remaining ones usually cause significant side effects, further limiting their use. Furthermore, MRSA and VRSA can form biofilms and communicate using quorum sensing in a bacterial cell density-dependent manner on medical devices and tissues, which protects them from antibiotics and the immune system, making infections even more difficult to eradicate. Thus, new approaches to combating *S. aureus* infections are being developed, involving different modes of action, such as the investigation of new efficient drug targets [6,7], the development of enzymbiotics against MRSA [8], engineered antimicrobials against multidrug-resistant pathogens [9], antimicrobial peptides [10] or vaccines [11].

Of interest, the genome of the *S. aureus* Mu50 genome (an MRSA strain with vancomycin-intermediate resistance; VISA) encodes five *N*-acetylglucosaminidases of the glycosyl hydrolase 73 (GH 73) family, which includes the following peptidoglycan hydrolases or autolysins (Atl): SAV2307 (AtlE), SAV1775 (SagB), SAV1052 (AtlA), SAV2644 (ScaH) and SAV0909 [12]. They are present in most *S. aureus* strains. According to the data known so far, all five *N*-acetylglucosaminidases are probably essential for the survival of *S. aureus* [12,13,14,15]. All autolysins are located on the outer surface of the bacterium and are therefore immediately accessible to potential antibacterial agents without needing to penetrate the bacterial membranes. They are bacteriolytic enzymes that play a key role in maintaining the equilibrium between bacterial peptidoglycan formation and degradation [16]. Peptidoglycan is a complex polymer that forms the rigid structure of the bacterial cell wall and consists of sequentially linked amino sugar units, namely *N*-acetylglucosamine (NAG) and *N*-acetylmuramic acid (NAM), which are further crosslinked with short peptides. As mentioned above, there is a dynamic balance between peptidoglycan assembly and degradation, and the maintenance of both processes is essential for bacterial growth, replication and, thus, survival. Peptidoglycan formation can be influenced by the inhibition of enzymes involved in its biosynthesis (e.g., muramyl (Mur) ligases, glycosyltransferases) or by the inhibition of enzymes involved in its degradation (autolysins) [17,18]. Autolysins are divided into three groups according to the type of covalent bond they cleave. Amidases cleave the bond between the NAM and the first amino acid residue of the polypeptide chain. Glycosidases cleave the bond between the sugars, whereby two subgroups are known: *N*-acetylglucosaminidases, which cleave the glycosidic bond between the NAG anomeric center and NAM; *N*-acetylmuraminidases and lytic transglycosidases, which cleave the glycosidic bond between the NAM anomeric center and NAG. Endopeptidases cleave the peptide bond between the amino acid residues of the polypeptide chain [19,20,21]. Inhibition of either group of enzymes involved in peptidoglycan formation or degradation has a potentially detrimental effect on bacterial infection, but the potential role of autolysin inhibitors as possible antibacterial agents still remains to be proven. To achieve this, potent and selective inhibitors of autolysins are needed.

**Figure 1:**
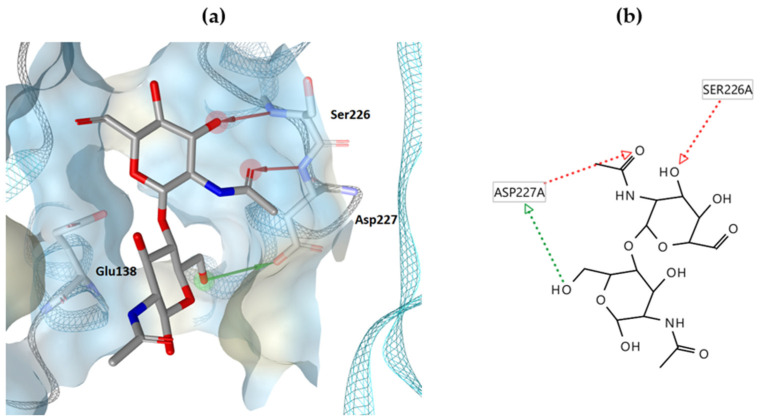
(**a**) 3D binding model of the NAG-NAM central unit on the AtlE surface (PDB ID: 4PI7); (**b**) 2D modeled interactions of the NAG-NAM central unit with AtlE (red residue represents the hydrogen bond acceptor, green residue represents the hydrogen bond donor).

However, the design of AtlE inhibitors is hampered by the fact that the structural and biochemical characterization of *S. aureus* autolysins is still poorly known and under investigation, as only a few studies have been published on AtlE [12,22,23]. So far, the most advanced studies have been performed on the major autolysin Atl [24,25,26]. The enzymes autolysin E, Mur A and Mur B, which are responsible for peptidoglycan metabolism, have been investigated in our previous research [27]. To date, the most accurate crystal structure of AtlE is the one co-crystallized with a peptidoglycan fragment containing three NAG-NAM disaccharide units (PDB ID: 4PI7) [12]. In this structure, the central NAG-NAM disaccharide binds near the catalytic Glu138, which is an important amino acid for the activity of the enzyme [12,22]. In addition, the disaccharide forms three other interactions with AtlE: a hydrogen bond (as a donor) with Ser226 and Asp227 and a hydrogen bond (as an acceptor) with Asp227 (Figure 1). Previously, various molecular modeling approaches have been used in the development of AtlE inhibitors. Fragments with micromolar AtlE-binding affinity were discovered by virtual screening of a virtual fragment library [28]. A chemical class of (phenylureido)piperidinyl benzamides as drug-like compounds with a binding affinity for AtlE in the low micromolar range was identified by virtual screening and further investigated by saturation-transfer difference (STD) NMR experiments [29].

In the present work, we evaluated iminosugars as potential binders for *S. aureus* AtlE. We investigated a set of iminosugars that have previously been studied for the inhibition of some other enzymes (α-glucosidase, β-glucosidase, α-galactosidase, β-galactosidase, naringinase, β-glucocerebrosidase, β-galactocerebrosidase) [30,31,32]. In this context, the compounds were docked in the AtlE crystal structure to rationalize binding to the active site of AtlE. Subsequently, the enzyme binding of the iminosugars was studied by a surface plasmon resonance (SPR) technique to evaluate the antibacterial activity of the compounds in vitro.

## 2. Results and Discussion

### 2.1. Iminosugar Dataset

Previous studies on glycosyl hydrolase family enzymes have identified new inhibitors of β-glucocerebrosidase [30,31,32]. β-glucocerebrosidase is an interesting drug target as it is responsible for the lysosomal storage disorder called Gaucher’s disease, a genetic disorder in which fat-laden Gaucher cells accumulate in areas such as the spleen, liver and bone marrow [33]. β-glucocerebrosidase is an enzyme with glucosylceramidase activity that cleaves the β-glycosidic bond of the glucocerebroside, which is similar to the NAG-NAM glycosidic bond. Various iminosugar derivatives as inhibitors of β-glucocerebrosidase have been developed due to their complementarity to the active sites of glycosidase and aspects of the relevant transition states in the hydrolysis processes catalyzed by glycosidases [34]. Since the hydrolysis mechanism is presumably similar to that of autolysins, we hypothesized that the iminosugars discovered by Dr. Pieters’ group [30,31,32] could also be potentially effective inhibitors of autolysins.

### 2.2. Molecular Docking Calculations

Our research started with structural information about the enzyme AtlE (PDB ID: 4PIA, 4PI7). We focused on the central NAG-NAM unit of PDB ID: 4PI7. Binding affinity was evaluated in silico with molecular docking using GOLD software 5.3. Successful validation of the molecular docking protocol was made by redocking of the NAG-NAM substrate in the binding pocket with an RMSD of 1.2 Å (Goldscore fitness = 5.2, Supplementary Table S1). Our dataset of eleven iminosugars was docked into the substrate binding grove with a radius of 12 Å around the central unit of the reference ligand NAG-NAM to obtain a binding model of potential interactions (Supplementary Table S1). Scoring was performed by reviewing the Goldscore scoring function and visual analysis with pharmacophore models in the binding site. The Goldscore fitness function was optimized for the prediction of ligand-binding positions. It includes factors such as hydrogen bond energy, van der Waals energy, metal interaction and ligand torsion strain [35]. As shown in Table 1, the values of the Goldscore fitness scoring function were in the range of 38.7 to 52.8. According to molecular docking scores, no exact correlation between the Goldscore fitness function and SPR results was observed. The compounds bound similarly to the NAG-NAM-binding pocket, with no major differences that became apparent upon further SPR analysis (see Section 2.4). Compound **1**, which had the best determined binding affinity (*K*_D_ = 19 µM), also had a high Goldscore fitness scoring function. Compounds **3** and **8** had lower Goldscore fitness values compared to compound **1**, which correlates with the measured *K*_D_ values.

**Figure 2:**
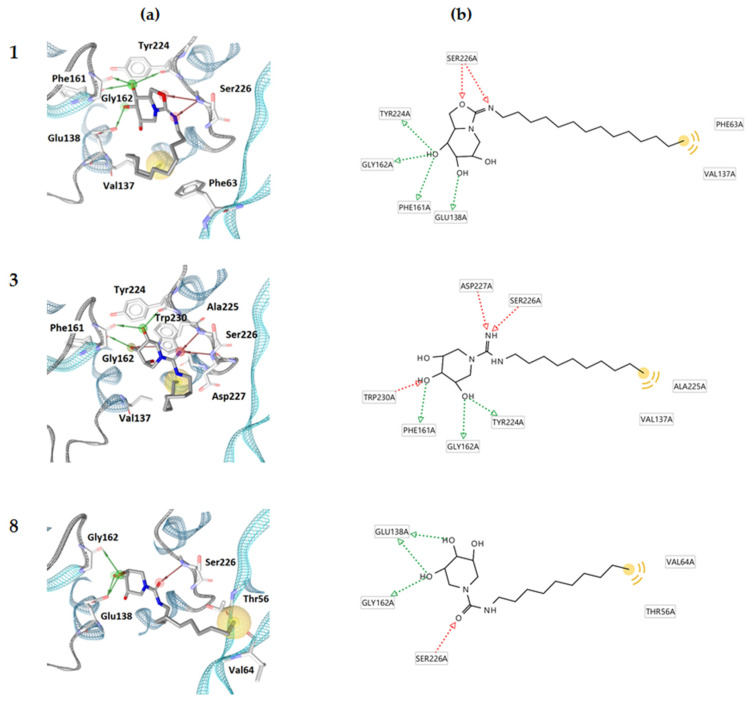
(**a**) 3D binding model of compounds **1**, **3** and **8** on the AtlE surface (PDB ID: 4PI7); (**b**) 2D modeled interactions of compounds **1**, **3** and **8** with AtlE (red residues represent the hydrogen bond acceptors, green residues represent the hydrogen bond donor, and yellow residues represent the hydrophobic interactions).

Visualization of the predicted binding positions (Figure 2) showed the formation of hydrogen bonds between compounds **1**, **3** and **8** and the amino acid residue Ser226, while compound **3** additionally formed a hydrogen bond with Asp227 and Trp230, with the functional groups of the compounds acting as a hydrogen bond acceptor. In the case of the same hydrogen bond with Gly162, the functional groups of compounds **1**, **3** and **8** acted as hydrogen bond donors, while compounds **1** and **3** shared two more common hydrogen bonds (Phe161, Tyr224). Compounds **1** and **8** also shared a common hydrogen bond with Glu138. The compounds did not form the same hydrophobic interactions. Compound **1** formed two interactions (Phe63, Val137), compound **3** had one (Ala225), and compound **8** showed two hydrophobic interactions with Val64 and Thr56. Compared to the binding pattern of NAG-NAM from the crystal structure (Figure 1), compounds **1**, **3** and **8** retained the binding pattern of one hydrogen bond with Ser226 and, thus, partially mimicked the binding of the peptidoglycan fragment NAG-NAM. The hydrophobic interactions of the saturated hydrocarbon chain seem to be less important than the H-bond interactions at the hydrophilic head of the compounds. The orientation of the heads for the three best compounds in the binding pocket is quite similar. Of interest, in previous research by Tibaut et al., only two small binding sites, around NAG-NAM and catalytic Glu138, composing a larger T-shaped binding site, were studied [28]. While similar to our case, Borišek et al. used one large binding site in the vicinity of NAG-NAM, where the common interactions of their compound 10 [29] and our compound **8** were observed: a hydrogen bond with Ser226 and a hydrogen bond with Gly162.

### 2.3. In Silico Physicochemical and Toxicity Assessment

The drug-like properties of the compounds were predicted using various prediction models and tools to evaluate their aqueous solubility, physiochemical, pharmacokinetic and toxicological properties. The physicochemical properties of the iminosugars were calculated with the SWISSADME tool [36]; the results are presented in Supplementary Table S2. All compounds have favorable physicochemical properties following drug-like rules. Furthermore, aqueous solubility was predicted using commercial prediction programs and in-house-developed models (m-id75, m-id90, m-id82, NN-A, NN-D) [37]. The results are listed in Supplementary Table S3. The predictions are in the range of moderate or poor solubility; the lower solubility was also observed experimentally as the addition of DMSO was necessary for the SPR measurement. Furthermore, a favorable pharmacological profile of hit compounds was also observed (Supplementary Table S4), with no crossing of the blood–brain barrier (BBB) and no cardiotoxicity due to inhibition of hERG (the human ether-a-go-go-related gene). For the toxicity assessment, hepatotoxicity, mutagenicity, carcinogenicity and other properties were analyzed (Supplementary Table S5). The results for hepatotoxicity show that the majority of compounds are not hepatotoxic. The compounds are also predicted to be non-nephrotoxic and non-mutagenic. The compounds appear to be carcinogenic, yet the reliability of the predictions is very low. The evaluation of endocrine disruption potential is also of interest, as adverse effects such as interference with the production, release, transport, metabolism, binding, action and secretion of natural hormones in the body may lead to undesirable interference with internal balance maintenance (homeostasis) or normal cell metabolism, fertility, behavior and development. In this regard, molecular docking was performed with Endocrine disruptome software (http://endocrinedisruptome.ki.si/, access date: 05 August 2024) to determine the binding of compounds on 14 different nuclear receptors: androgen receptor, estrogen receptors α and β, glucocorticoid receptor, liver X receptors α and β, mineralocorticoid receptor, peroxisome proliferator-activated receptor α, β/δ and γ, progesterone receptor, retinoid X receptor α and thyroid receptor α and β. All hit compounds from our study have a favorable endocrine disruption profile, as represented in Supplementary Table S5.

### 2.4. Surface Plasmon Resonance (SPR) Analysis

The potential inhibitory effect of compounds that bind to AtlE was measured using surface plasmon resonance (SPR). The protein was covalently attached to the surface of the CM5 sensor chip (5200 response units (RU)). The compounds were prepared as 20 mM stock solutions in DMSO and diluted with HEPES running buffer with 2.5% DMSO. Some compounds were less soluble, which was also predicted by the in silico programs and QSAR models for aqueous solubility (see Supplementary Table S3).

**Figure 3:**
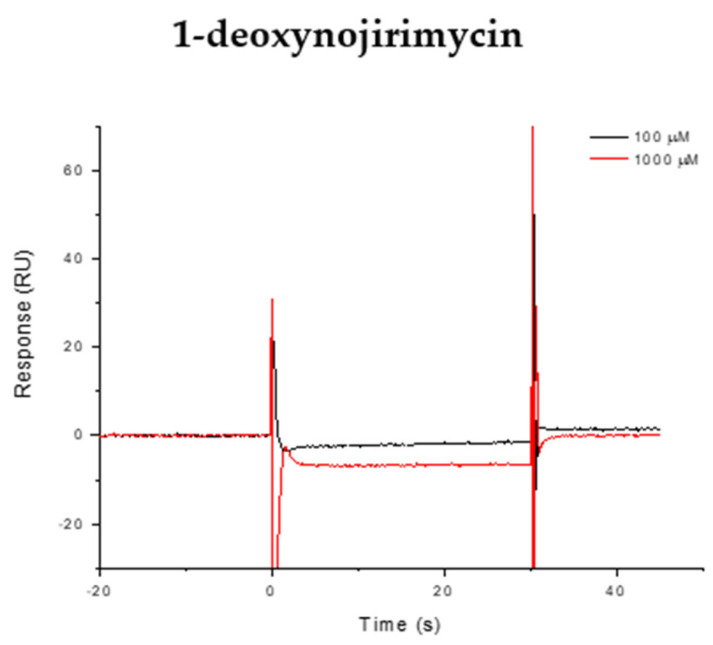
Representative SPR sensorgram (response curves) for compound 1-deoxynojirimycin at two different concentrations.

The binding of iminosugars was recorded and analyzed using the Biacore T200 3.2.1 software v3.2.1 Evaluation (for results, see Table 1). First, we tested 1-deoxynojirimycin (DNJ) at two concentrations (100 µM and 1 mM) and recorded no binding of AtlE (Figure 3). In addition, we observed that sugar mimetics with an amino group (nojirimycin and analogues) did not bind AtlE even up to 1000 µM but required a longer lipophilic aglycone (*n*-nonyl fragment) for stronger binding. Therefore, for further testing of AtlE binding, the iminosugar derivatives with a lipophilic tail were chosen (1,5-dideoxy-1,5-imino-D-xylitol derivatives (DIX)).

**Figure 4:**
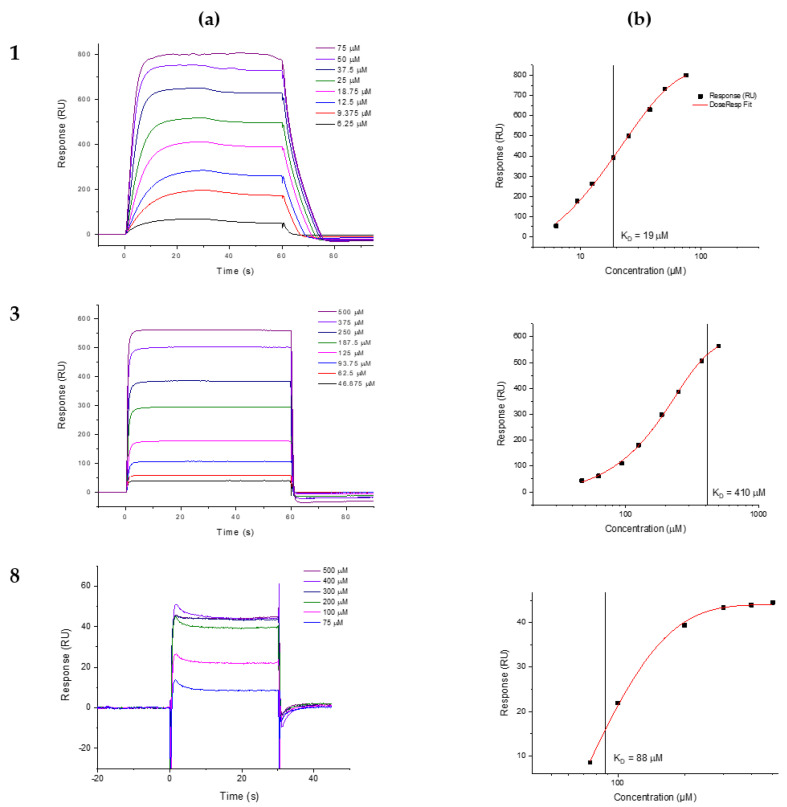
(**a**) Representative SPR sensorgrams (response curves) and (**b**) representative saturation curves with evaluated *K*_D_ for compounds **1**, **3** and **8** at different concentrations. SPR analysis of compound **1**, **3** and **8** interactions with the immobilized AtlE. Compounds were injected across immobilized AtlE in serial dilutions for 60 s at a rate of 30 mL/min, and the dissociation was followed for 50 s. Sensorgrams are shown along with the apparent equilibrium dissociation constant (*K_D_*) determined from the response curves as a function of the compound concentration injected across AtlE. *K_D_* values are the mean ± standard deviation of three titrations. The data were fitted to the steady-state affinity binding model.

Compounds **7**, **9**, **10** and **11** exhibited binding affinity in the millimolar range to the AtlE enzyme (*K*_D_ > 1000 µM). Other compounds (**2**, **4**, **5**, **6**) showed a linearly increasing response (LR) with increasing concentration (Figure S1), while the *K*_D_ could not be determined due to the lack of saturation. Three compounds, **1**, **3** and **8,** were identified as AtlE binders. Two of them, **1** and **3,** were titrated at eight different concentrations, while compound **8** was titrated firstly at six different concentrations (Figure 4a) and secondly at seven concentrations (Figure S2). The equilibrium dissociation constant *K*_D_ was estimated using the 1:1 steady-state affinity binding model, with the graphs showing the response units as a function of inhibitor concentration (Figure 4b). The *K*_D_ value for compound **1** was 19 ± 3.7 μM and 410 ± 4.9 μM for compound **3**, while the *K*_D_ value for compound **8** was 88 ± 4.2 μM. The maximal theoretical response was calculated using the molecular masses of AtlE and the compounds, respectively. A higher maximal response than expected was measured for compounds **1** and **3**. Due to the amphiphilicity of the compounds with a hydrophilic sugar unit in the head and a hydrophobic fatty acid residue in the tail, we can assume that both compounds can form oligomeric micelles. To prevent the formation of micelles, we added a surfactant P20 to the running buffer, but the maximum theoretical binding to AtlE was not reached for compounds **1** and **3**. This brings into question the true nature of their binding and should be verified by another independent method. The only specific AtlE binder that we can confirm with certainty appears to be compound **8**, for which we have achieved the expected maximal theoretical response.

On the other hand, in the case of the linear responses, we cannot know whether they are indeed AtlE non-binders. We can assume that compounds **2**, **4**, **5** and **6** also form micelles and are, therefore, not present in solution as monomolecular species. Interestingly, compound **1**, the bicyclic isourea analogue of DNJ, and compound **3**, the guanidino analogue of DIX, inhibit human recombinant β-glucocerebrosidase with IC_50_ values in the low nanomolar range, whereas no binding of this enzyme was observed for compound **8**, the urea analogue of DIX, indicating that **8** can be considered a selective AtlE binder [21,22]. Moreover, compound **1** has an alkyl chain of fourteen carbon atoms, while compounds **3** and **8** both have an alkyl chain of ten carbon atoms. As can be seen, molecules with longer alkyl chains with saccharide sequences are preferred for AtlE. Thus, iminosugars with a partially conserved sugar structure, a cationic center, or at least a ureido structure, and a lipophilic aglycone were able to bind to AtlE. In a study by Tibaut et al., a *K*_D_ of 228 μM was determined for the compound fragment F1 [28]. In addition, the study by Borišek et al. identified ten compounds of (phenylureido) piperidinyl benzamides as the first reported non-substrate-like inhibitors for AtlE, with *K*_D_ values ranging from 1.9 to 177 μM [29]. However, compound **8**, which showed the best specific binding of AtlE in our study (*K*_D_ = 88 μM), was observed in previous studies as an inhibitor of glucosidase but not as an inhibitor of β-glucocerebrosidase [31] (Supplementary Table S7).

### 2.5. Minimal Inhibitory Concentration (MIC)

The minimal inhibitory concentrations (MICs) were determined against the bacterial strains *S. aureus* (ATCC 29213) and *E. coli* (ATCC 25922), and the results are presented in Table S6. The antimicrobial susceptibility against *E. coli* ATCC 25922 served as a negative control, as *E. coli* does not have autolysin or its related enzyme. Seven compounds, labeled **1**, **2**, **4**, **5**, **6**, **7** and **9**, showed modest antibacterial activities against *S. aureus* ATCC 29213. The most potent compounds were **6** and **1**, where MIC values were ≤4 and 8 µM for *S. aureus*, respectively.

## 3. Materials and Methods

### 3.1. Dataset

The compound 1-deoxynojirimycin was used as the starting point for the AtlE enzyme-binding assay. Then, a set of iminosugars (n = 16) was used for the molecular docking approach. In vitro testing consisted of eleven compounds, where two compounds, **1** and **2**, were bicyclic isoureas derived from 1-deoxynojirimycin (DNJ) analogue with *N*^G^-substituted bicyclic isourea [30]. The 1,5-dideoxy-1,5-imino-d-xylitol (DIX) analog with *N*^G^-substituted guanidine was **3**, **4**, **5** and **6**, while the DIX analog with *N*^G^-substituted urea was **7**, **8**, **9** and **10** [31]. Compound **11** was orthoester functionalized with DIX [32].

### 3.2. Molecular Docking Calculations

The molecular docking experiments were performed using the GOLD software [38,39] and the crystal structure of *S. aureus* autolysin E (AtlE) in complex with 3 units of disaccharide NAG-NAM (PDB ID: 4PI7) [12]. In the first step, the validation of the GOLD docking tool was performed [40] by redocking the native ligand NAG-NAM molecule into its binding site. The binding site was defined as a 12 Å radius around the central unit of the reference ligand, NAG-NAM. The observed heavy-atom root-mean-square deviation (RMSD) of the obtained docked poses versus the original NAG-NAM position were within the accepted limits (RMSD ≤ 2.0 Å), and the Goldscore scoring function was used. The same scoring function and described docking settings were used for the molecular docking calculations of all compounds. The GOLD genetic algorithm parameters (population size = 100, selection pressure = 1.1, number of operations = 100,000, number of islands = 5, migrate = 10, mutate = 95, niche size = 2, crossover = 95.42) were used. Each molecule was docked 10 times into the binding site; further, all docking calculations were visualized and geometrically analyzed in LigandScout [35].

### 3.3. In Silico Physicochemical and Toxicity Assessment

The calculation of physicochemical, water solubility, pharmacokinetic and toxicological properties for ligands was made using a variety of different in silico models and tools. The programs used were VEGA [41], TEST [42], SwissADME [36], pkCSM [43], admetSAR [44,45], Vienna LiverTox [46] and Endocrine disruptome [47].

### 3.4. Surface Plasmon Resonance (SPR) Analysis

Surface plasmon resonance (SPR) experiments were performed at 25 °C using a Biacore T200 (Biacore, GE Healthcare, Uppsala, Sweden) instrument with Series S sensor chip CM5 (GE Healthcare). AtlE was produced as previously described [12] and immobilized on the second flow cell of the chip by the amino coupling method with HEPES running buffer (10 mM HEPES, 150 mM NaCl, 3 mM EDTA, 0.005% surfactant P20, pH 7.4). The carboxymethylated dextran layer was activated with a 720 s pulse of 0.4 M EDC (1-ethyl-3-(3-dimethylethylaminopropyl)-carbodiimide) and 0.1 M NHS (*N*-hydroxysuccinimide) in a 1:1 ratio. AtlE, diluted to a final concentration of 50 µg/mL in 10 mM sodium acetate (pH 6.3), was injected to reach a final immobilization level of 5200 RU. The rest of the surface was deactivated with a 600 s injection of ethanolamine. The first flow cell served as a reference cell for the subtraction of nonspecific binding and was activated with EDC/NHS and deactivated with ethanolamine. For screening, the HEPES running buffer supplemented with 2.5% (*v*/*v*) DMSO (dimethyl sulfoxide) (Merck) and 0.1% BSA (bovine serum albumin) was used. In the first step, selected compounds were tested at two different concentrations: 100 μM and 1 mM. Each compound was injected at a flow rate of 5 μL/min for 30 s, and the dissociation was monitored for 30 s, where the buffer flow was used to stabilize the surface after each injection. Compounds showing binding were tested at different concentrations, depending on their solubility, in three parallel titrations. Regeneration was provided with 2.5 mM NaOH for 8 s.

Sensorgrams have been reviewed using Biacore T200 software v3.2.1 Evaluation (Biacore, GE Healthcare, Uppsala, Sweden). *K_D_* values were determined using Biacore T200 software v3.2.1 Evaluation by fitting the data to a 1:1 steady-state affinity model.

### 3.5. Minimal Inhibitory Concentration (MIC)

Antimicrobial testing was carried out by the broth microdilution method in a 96-well plate format following the CLSI guidelines [48] and European Committee for Antimicrobial Susceptibility Testing (EUCAST) recommendations [49]. Bacterial suspension of a specific bacterial strain equivalent to 0.5 McFarland turbidity standard was diluted with cation-adjusted Mueller Hinton broth to obtain a final inoculum of 10^5^ CFU/mL. Compounds dissolved in 20% DMSO and inoculum were mixed together and incubated for 20 h at 35 °C. After incubation, the minimal inhibitory concentration (MIC) values were determined by visual inspection as the lowest dilution of compounds showing no turbidity. The MICs were determined against *S. aureus* (ATCC 29213) and *E. coli* (ATCC 25922) bacterial strains. Tetracycline was used as a positive control on every assay plate, showing MICs of 0.5 µg/mL and 1 µg/mL for *S. aureus* and *E. coli*, respectively.

## 4. Conclusions

In this study, we evaluate iminosugars as potential antibacterial agents against *S. aureus* by inhibiting autolysin E (AtlE). In silico and in vitro assays were performed on eleven iminosugar compounds. The results of molecular docking calculations, pharmacophore modeling and visual analysis were further investigated using surface plasmon resonance (SPR). We found that the orientation of the heads of all three best-hit (**1**, **3**, **8**) pockets is quite similar. All three compounds exhibit the same hydrogen bond donor binding with Ser226 and hydrogen bond acceptors with Gly162. Iminosugars have relatively favorable physicochemical properties consistent with drug-like rules and a favorable pharmacological profile. As expected, 1-deoxynojirimycin did not inhibit AtlE, as measured by SPR. Of the iminosugars tested by SPR, compounds **1**, **3** and **8** show binding to AtlE in the micromolar range. The strongest compound among the SPR response curve hits was compound **1**, with a *K*_D_ of 19 μM. The *K*_D_ value for the next most potent compound **8** was 88.1 μM, while compound **3** had a *K*_D_ of 410 μM. A higher than expected maximum response was measured for compounds **1** and **3**, which leads us to assume that both compounds can form oligomeric micelles due to their amphiphilicity with a hydrophilic sugar moiety in the head and a hydrophobic fatty acid residue in the aglycone. For compound **8**, for which the expected maximum theoretical response was not exceeded, specific binding to the AtlE enzyme can be evaluated. Antimicrobial susceptibility testing of compounds on *S. aureus* showed that the iminosugars likely have an antibacterial effect by inhibiting the AtlE enzyme. Autolysins are not yet validated as therapeutic targets, so such studies are a valuable effort to approach their validation. In any case, this research is a step forward in the development of new, urgently needed antibacterial agents. The results presented here are a good starting point for further optimization of AtlE inhibitors.

## Figures and Tables

**Table 1:** Chemical structures of iminosugar derivatives with molecular docking scores (Goldscore fitness) and equilibrium dissociation constants (K_D_) obtained by SPR measurements.

ID	Structure	X	Goldscore Fitness	*K*_D_ (µM)
1 (14 d [30])	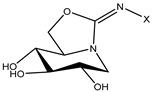	C14	52.1	19
2 (14 e [30])		CH_2_CON(C10)_2_	51.1	LR
3 (14 b [31])	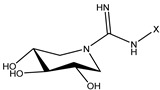	C10	44.5	410
4 (14 c [31])		C12	51.3	LR
5 (14 d [31])		C14	42.4	LR
6 (14 e [31])		CH_2_CON(C10)_2_	52.8	LR
7 (17 a [31])	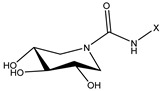	C8	38.7	>1000
8 (17 b [31])		C10	46.7	88
9 (17 c [31])		C12	46.7	>1000
10 (17 d [31])		C14	47.5	>1000
11 (10 [32])	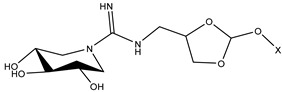	C4	44.1	>1000

LR means linearly increasing concentration response. **1** and **2** are bicyclic isourea derivatives of DNJ [30]; **3**, **4**, **5**, **6** are guanidino derivatives of DIX [31]; **7**, **8**, **9**, **10** are urea derivatives [31]; **11** is an orthoester derivative of DIX [32].

## Data Availability

The data presented in this study are available on request from the corresponding authors.

## Supplementary Materials

The following supporting information can be downloaded at https://www.mdpi.com/article/10.3390/antibiotics13080751/s1, Table S1: Chemical structures of iminosugars with 2D modeled interactions of compounds with AtlE, 3D binding model of compounds on the AtlE surface (PDB ID: 4PI7) and Kd values from SPR; Table S2: Physicochemical properties of iminosugars; Table S3: Water solubility properties of iminosugars; Table S4: Pharmacokinetics properties of iminosugars; Table S5: Toxicity properties of iminosugars; Table S6: Antimicrobial activity of iminosugars against *S. aureus* (ATCC 29213) and *E. coli* (ATCC 25922) bacterial strains; Table S7: Glycosidase inhibition values of iminosugars; Figure S1. Representative SPR sensorgrams of iminosugars that show a linearly increasing response with increasing concentration. Figure S2. Representative SPR sensorgram of compound **8** (2nd dilution).
